# Strong phenotypic divergence in spite of low genetic structure in the endemic Mangrove Warbler subspecies (*Setophaga petechia xanthotera*) of Costa Rica

**DOI:** 10.1002/ece3.5826

**Published:** 2019-11-19

**Authors:** Tania Chavarria‐Pizarro, Juan Pablo Gomez, Judit Ungvari‐Martin, Rachael Bay, Michael M. Miyamoto, Rebecca Kimball

**Affiliations:** ^1^ Department of Biology University of Florida Gainesville FL USA; ^2^ Department of Biology Ludwig Maximilian University of Munich Munich Germany; ^3^ Departamento de Química y Biología Universidad del Norte Barranquilla Colombia; ^4^ Department of Evolution and Ecology University of California Davis CA USA

**Keywords:** ecological genomics, environmental gradient, gene flow, Mangrove Warbler, phenotypic divergence, *P*_ST_–*F*_ST_

## Abstract

Despite the enormous advances in genetics, links between phenotypes and genotypes have been made for only a few nonmodel organisms. However, such links can be essential to understand mechanisms of ecological speciation. The Costa Rican endemic Mangrove Warbler subspecies provides an excellent subject to study differentiation with gene flow, as it is distributed along a strong precipitation gradient on the Pacific coast with no strong geographic barriers to isolate populations. Mangrove Warbler populations could be subject to divergent selection driven by precipitation, which influences soil salinity levels, which in turn influences forest structure and food resources. We used single nucleotide polymorphisms (SNPs) and morphological traits to examine the balance between neutral genetic and phenotypic divergence to determine whether selection has acted on traits and genes with functions related to specific environmental variables. We present evidence showing: (a) associations between environmental variables and SNPs, identifying candidate genes related to bill morphology (BMP) and osmoregulation, (b) absence of population genetic structure in neutrally evolving markers, (c) divergence in bill size across the precipitation gradient, and (d) strong phenotypic differentiation (*P*_ST_) which largely exceeds neutral genetic differentiation (*F*_ST_) in bill size. Our results indicate an important role for salinity, forest structure, and resource availability in maintaining phenotypic divergence of Mangrove Warblers through natural selection. Our findings add to the growing body of literature identifying the processes involved in phenotypic differentiation along environmental gradients in the face of gene flow.

## Graphical abstract

We used single nucleotide polymorphisms (SNPs) and morphological traits to examine the balance between neutral genetic and phenotypic divergence to determine whether selection has acted on traits and genes with functions related to specific environmental variables in the endemic subspecies of Mangrove Warbler in Costa Rica along an environmental gradient

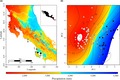

## 1 INTRODUCTION

Populations distributed along environmental gradients provide excellent systems to study the counteracting effects of natural selection and gene flow on population trait divergence at relatively small geographic scales (Bertrand et al., 2016; Doebeli & Dieckmann, 2003; Milá, Warren, Heeb, & Thébaud, 2010; Postma & Noordwijk, 2005; Richardson, Urban, Bolnick, & Skelly, 2014; Schluter, 2009; Seeholzer & Brumfield, 2018; Smith et al., 2005). If environmentally mediated divergent selection is strong enough to counteract the homogenizing effects of gene flow, trait variability could persist through time (Milá et al., 2010; Richardson et al., 2014; Schluter, 2009; Smith et al., 2005). This ecologically driven trait divergence could result in speciation if traits subject to natural selection secondarily influence sexual traits (Schluter, 2001). Since in this model, there is no physical separation between incipient species, there is still potential for interbreeding and gene flow during the speciation process (Bolnick & Fitzpatrick, 2007; Foote, 2018). For these reasons, it is essential to understand the conditions and processes under natural selection that can drive ecological divergence and reproductive isolation in continuously distributed populations, especially during early stages of formation of biological diversity (Bolnick & Fitzpatrick, 2007; Cheviron, Connaty, McClelland, & Storz, 2014).

It is a difficult challenge in nature, however, to assess the frequency and factors that facilitate the process of divergence in the presence of gene flow (Bolnick & Fitzpatrick, 2007; Hendry, 2009; Maan & Seehausen, 2011; Smadja & Butlin, 2011). Trait variability among populations could be detected using a variety of methods, including the study of morphological characteristics (Cheviron & Brumfield, 2009; Eroukhmanoff, Hermansen, Bailey, Sæther, & Sætre, 2013; Funk, Nosil, & Etges, 2006; Maley, 2012; Milá et al., 2010; Seeholzer & Brumfield, 2018), genomewide trait and environment associations (Whitehead & Crawford, 2006), study of outlier loci (Bay et al., 2018; Schweizer et al., 2016), and detecting transcriptional plasticity (Cheviron et al., 2014). Studies that use a combination of genomic and phenotypic approaches can lead to a better understanding of how trait variability is maintained even in the absence of substantial geographic or behavioral reproductive isolation (Charmantier, Doutrelant, Dubuc‐Messier, Fargevieille, & Szulkin, 2016).

**Figure 1:**
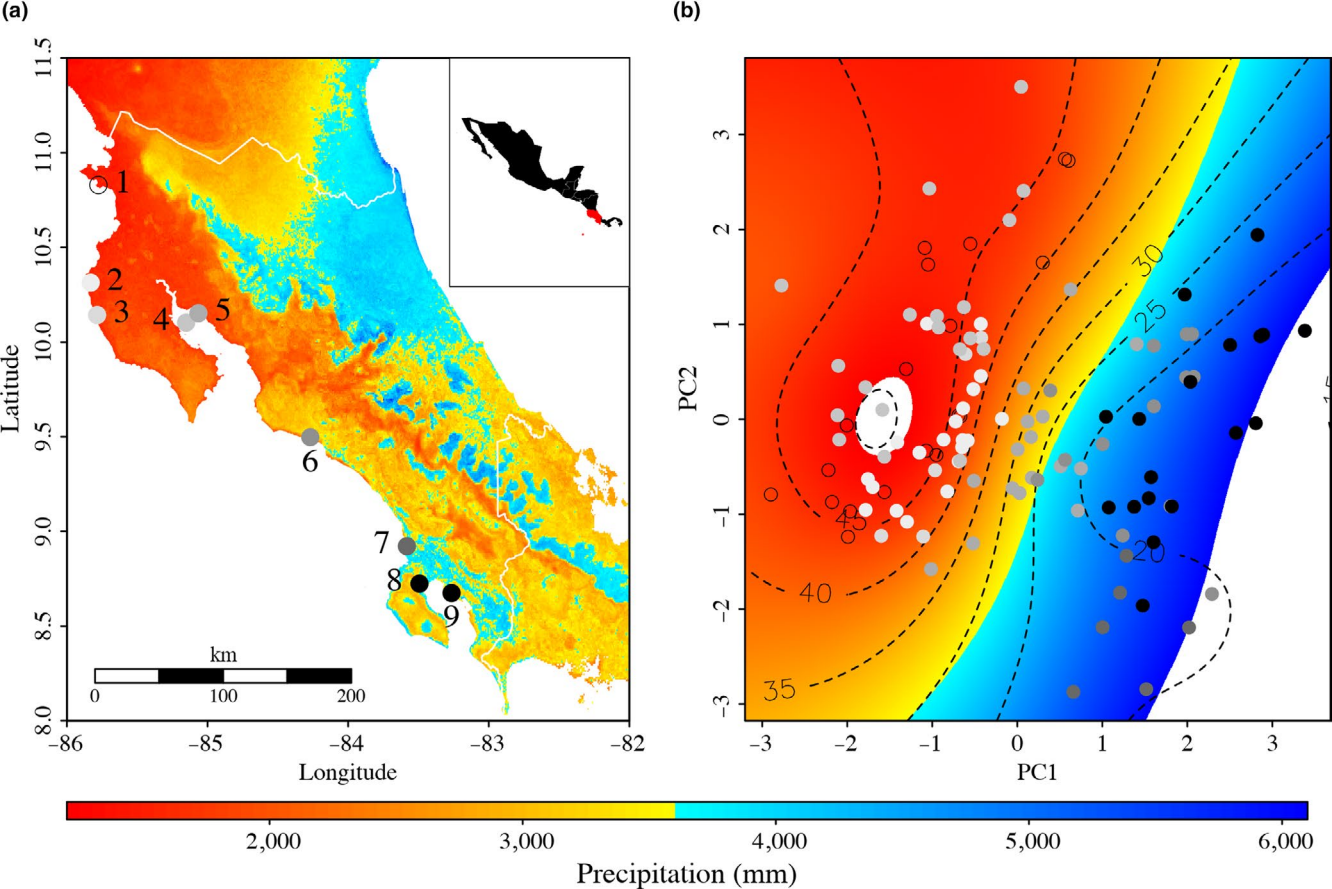
(a) Map showing the sampling locations of Mangrove Warbler populations and the precipitation gradient. The salinity gradient correlates with the precipitation gradient such that drier sites have higher salinity levels. Each individual sampling site is noted by different colors shown in the map. Population names correspond to 1 = Naranjo, 2 = Junquillal, 3 = Chira, 5 = Chomes, 6 = Tarcoles, 7 = Sierpe, 8 = Osa, 9 = Golfito. (b) Principal component analysis showing the morphological distribution of individuals along precipitation gradient in Costa Rica. Colors show average precipitation gradient and dashed lines with black numbers represent the average salinity levels

The Pacific coast of Costa Rica presents a strong precipitation and salinity gradient in which yearly rainfall varies between 1,000 mm in the north and ~6,000 mm in the south creating a salinity gradient that has a strong influence on mangrove forest structure (Figure 1a). Where rainfall is low, and salinity is high, canopy level rarely exceeds 20 m in height, while in areas with high rainfall and low salinity canopy can exceed 35 m (Jiménez, 1990). Several bird species are distributed along the gradient from which Mangrove Warbler (*Setophaga petechia xanthotera*) is restricted to the mangrove habitat. The environmental gradient of the Pacific coast of Costa Rica, then, has the potential to influence the divergence of traits involved in physiology, foraging, and possibly behavior of this insectivorous habitat specialist bird.

For example, insects accumulate excess salt in their exoskeleton in high salinity environments (Bradley, 2008). Salt regulation is particularly problematic for passerine species because they lack salt glands. Thus, differences in salinity and water availability should promote divergence of osmoregulatory genes or their expression, to help individuals deal with salt regulation and water loss at environmental extremes (Sabat, Maldonado, Rivera‐Hutinel, & Farfan, 2004; Sugiura, Aste, Fujii, Shimada, & Saito, 2008). Additionally, differences in forest structure driven by the abiotic environment could influence size distribution of insect prey (Janzen & Schoener, 1968). Other characteristics of the forest such as understory density and overall forest interior structure are also affected by precipitation and salinity. Specifically, bill morphology might change along the gradient as a response to the change in overall resource size distribution (Grant & Grant, 2011), while wing, tail, and tarsus morphology could be influenced by the forest structure as these traits are directly related to flight performance and maneuverability (Milá et al., 2010; Pennycuick, 1968; Ricklefs, 2012; Thomas, 1996). Consequently, abiotic environment, resources, and forest structure can all have an impact on ecophysiological and morphological traits of Mangrove Warbler.

Prior to this study, we posited that Mangrove Warbler populations would have high levels of gene flow based on two facts: (a) There are no strong geographic barriers (e.g., mountains ranges, large river basins) to isolate the Mangrove Warbler populations along the gradient and (b) high levels of gene flow have been previously documented among populations of the Galapagos and Coco's island warbler (*Setophaga petechia aeurolea*) in spite of their strong geographic isolation (Chaves, Parker, & Smith, 2012). For these reasons, Mangrove Warblers on the Pacific coast of Costa Rica should provide an excellent system to study the effect of the environment on trait divergence with potential gene flow.

Studies that investigate the role of environmental gradients on adaptive variation in birds largely focus on divergence along elevational gradients (Cheviron & Brumfield, 2009; Milá, Wayne, Fitze, & Smith, 2009; Schluter, 2001; Seeholzer & Brumfield, 2018; Vines & Schluter, 2006). Few studies have attempted to determine the role of precipitation and salinity gradients in birds phenotypic variability (Bay et al., 2018), and even fewer studies have focused on understanding the genomic patterns behind trait variation along environmental gradients (Cheviron et al., 2014). In this study, we obtained samples from nine populations of Mangrove Warbler distributed along a steep precipitation gradient on the Pacific coast of Costa Rica to explore the role of the environmental gradient on phenotypic and genetic divergence. Our main objectives were to (a) identify genomic signals of selection, (b) determine levels of neutral genetic divergence among these populations (*F*_ST_), (c) estimate levels of phenotypic divergence along the environmental gradient (phenotypic structure: *P*_ST_), and (d) determine the balance between genetic and phenotypic divergence.

## 2 METHODS

### 2.1 Mangrove Warbler sampling

Between 2013 and 2015, we visited nine localities distributed along the precipitation gradient on the Pacific coast of Costa Rica (Figure 1a). At these localities, we captured 115 adult male and female Mangrove Warblers (*S. p. xanthotera*). For every individual captured, we measured body mass, bill length (from the nares to the tip) and height (top and bottom bill at the nostrils point), tarsus (from the inner bend of the tibio‐tarsal articulation to the base of the toes), flattened wing cord length (bend of the wing to the tip of the longest primary feathers), and tail length (base of the tail to the tip of the longest feathers). These morphological traits were measured with a caliper (±0.005) except for body weight which was measured with a 100 g (±0.01) analog scale. These traits were chosen because they are expected to respond to ecological differences in habitat (Eroukhmanoff et al., 2013; Grant & Weiner, 1999; Maley, 2012; Ricklefs, 2012). We also obtained blood samples for genetic analyses, which were stored in lysis buffer (0.1 M Tris–HCl, 0.1 M EDTA, 0.01 M NaCl, 2% SDS).

We mapped all mangrove localities reported for Costa Rica using recent satellite images available at Centro Nacional de Alta Tecnología (CENAT). The nine populations chosen along the environmental gradient were selected based on their accessibility. Although we considered including Atlantic populations in our sampling, we were not able to find any Mangrove Warbler individual at the Caribbean during the time of our study. Using digitized maps, we calculated the Euclidean and coastal distances among sampling populations. Euclidean distance refers to the straight line between sites (so might involve flying over water) while coastal distance is the distance of shoreline between two sites along the coast (assuming birds would not fly over water). Sampling sites were between 15 and 345 km apart using Euclidean distance and between 15 and 2,584 km apart using coastal distance.

### 2.2 Environmental variables and habitat classification

To characterize the environment in each of the populations sampled, we used georeferenced environmental data sets from the Meteorological Institute of Costa Rica, WorldClim (Fick & Hijmans, 2017), and a database from the University of Costa Rica Marine Investigation Center (CIMAR). We gathered data for a set of eight environmental variables: mean annual temperature (BIO1), mean diurnal temperature range (BIO2), temperature seasonality (BIO4), annual precipitation (BIO12), precipitation of the wettest month (BIO13), precipitation of the driest month (BIO14), precipitation seasonality (BIO15), elevation (SRTM), and mean water salinity (ppt)(CIMAR). However, since precipitation variables (BIO12, BIO13, BIO14, BIO15) were highly correlated (*R* > 0.88 among the four variables), and we considered that precipitation was the principal factor affecting conditions along the gradient (since precipitation affects soils salinity levels which in turn influence the forest structure), consequently, we used only annual precipitation (BIO12) to describe the environmental gradient and for analysis of our morphological and genotypic data (Table 1).

**Table 1:** Coordinates, habitat, and a mean annual precipitation (BIO12), mean salinity levels (ppm), canopy height (m) of localities sampled and number of individuals by population, used in the genetic (SNPs) and morphological analyses

Population	Locality	Coordinates	Habitat	BIO12	Salinity (ppm)	Canopy (m)	SNPs	Morphology
1	Naranjo	10.77, −85.66	Dry‐high salinity	1,300	55	<15	5	15
2	Tamarindo	10.81, −85.83	Dry‐high salinity	1,500	45	<15	12	20
3	Junquillal	10.15, −85.80	Dry‐high salinity	1,500	45	<15	3	5
4	Chira	10.08, −85.11	Dry‐high salinity	1,700	47	<15	2	3
5	Chomes	10.06, −84.95	Dry‐high salinity	1,700	41	<15	13	19
6	Tarcoles	9.78, −84.64	Wet‐low salinity	3,200	25	15–35	8	16
7	Sierpe	8.88, −83.60	Wet‐low salinity	4,000	17	>35	9	14
8	Osa	8.69, −83.47	Wet‐Low Salinity	5,900	18	>35	3	5
9	Golfito	8.64, −83.17	Wet‐Low Salinity	6,000	19	>35	13	18

### 2.3 Collection of genetic data and identification of SNPs

To estimate the variation in genetic structure and patterns of gene flow among populations of Mangrove Warbler distributed along the environmental gradient, we obtained SNPs using double‐digest RADseq (ddRADseq). We first extracted DNA from the blood samples using the Qiagen PureGene DNA Isolation kit. DNA extractions were quantified using a NanoDrop ND8000 spectrophotometer and a Qubit 2.0 fluorometer with the DNA HS assay kit (Life Technologies) and checked for DNA quality on an agarose gel to select samples with appropriate DNA concentration (>20 ng/µl) and quality. From the 115 individuals collected and for which we had morphological data, we only succeeded in extracting DNA, constructing libraries, and obtaining reliable genetic data from 68 of them (Table 1), following the method proposed by Peterson, Weber, Kay, Fisher, and Hoekstra (2012), using EcoRI and MspI. Each individual RAD library was ligated to a unique molecular identifier using one of four types of DNA barcodes (either 8, 9, 10, 14 bp in length). Individuals were pooled together, fragments were subject to size selection to produce a mean fragment length of 250–440 bp, and then sequenced on a single Illumina NextSeq 500 run. The sequenced reads were quality‐filtered, and individual barcode information was removed using the process_radtags program in STACKS (1.46) (Catchen, Amores, Hohenlohe, Cresko, & Postlethwait, 2011; Catchen, Hohenlohe, Bassham, Amores, & Cresko, 2013). Afterward, PCR clone sequences were eliminated with “clonefilter” in STACKS v1.30 (Catchen et al., 2011). We chose STACKS because it is a specialized software pipeline for building loci from short‐read sequences with restriction enzyme‐based data. We aligned the samples based on the reference genome available for Yellow Warbler (*Setophaga petechia petechia*) (Bay et al., 2018) using BWA‐MEM V.0.7.9a (Li & Durbin, 2009). This allowed for additional positioning information and facilitated detecting rare allele variants (Peterson et al., 2012) that are often removed from de novo assemblies, as they can be confounded with sequencing errors.

Subsequently, we used PSTACKS, CSTACKS, and SSTACKS (Catchen et al., 2011, 2013; Paris, Stevens, & Catchen, 2017) to identify SNPs. Parameters for these alignments included a terminal threshold of 500, a maximum number of mismatches allowed (*M* = 5), a minimum stack depth of three (*m* = 3) among reads with potentially variable sequences (Paris et al., 2017), and an indel penalty of 2 (Catchen et al., 2011). In the population's module of STACKS and following consecutive filtering steps, we first retained RAD tags with a minimum stack depth (*m*) of 20 and a maximum stack depth of 100. This step removed SNPs genotyped with too low coverage (*m* < 20) to be accurately called as well as SNPs genotyped with too high coverage (*m* > 100) that might reflect repetitive regions. Then, we retained SNPs genotyped in at least 80% of the individuals and 80% of the sampling locations and excluded markers showing heterozygosity >0.50 within samples (Hohenlohe, Amish, Catchen, Allendorf, & Luikart, 2011). We also removed markers out of Hardy–Weinberg equilibrium (*p*‐value = .01) at more than 60% of the locations. SNPs with more than 30% missing data were also eliminated. Finally, we removed SNPs with very low frequency (MAF < 0.05), as these can create biases in quantifying genetic connectivity and should be removed when inferring demographic processes (Roesti, Hendry, Salzburger, & Berner, 2012). The number of SNPs kept after each filtering step can be found at the Appendix S1 in Table S1. Finally, we used the "Populations" module in STACKS to obtain individual genotypes per populations.

### 2.4 Identification of outlier loci and correlation with the environment

Single nucleotide polymorphisms potentially under balancing and divergent selection should be removed when assessing genetic connectivity (gene flow) among populations (Beaumont & Nichols, 1996). We searched for loci with a level of population differentiation exceeding neutral expectations using two *F*_ST_ based outlier analyses. First, we used the software OUTFLANK (Whitlock & Lotterhos, 2015), which calculates a likelihood based on a trimmed distribution of *F*_ST_ values to infer the distribution of *F*_ST_ for neutral markers. OUTFLANK was run with default options (LeftTrimFraction = 0.05, RightTrimFraction = 0.05, Hmin = 0.1, 19) and identified outlier SNPs across the nine populations based on the *Q*‐threshold of 0.05. Second, we detected outlier SNPs with BAYESCAN v. 2.1 (Foll & Gaggiotti, 2008) that estimates population‐specific *F*_ST_ coefficients using the Bayesian method and uses a cutoff based on the mode of the posterior distribution to detect SNPs under selection (Foll & Gaggiotti, 2008). SNPs with a posterior probability over 0.95 were considered as outliers, after running 100,000 iterations on all samples together (i.e., not pairwise, with remaining default parameters). We specified a ‘prior’ odd of 10,000, which set the neutral model being 10,000 times more likely than the model with selection to minimize false positives (Whitlock & Lotterhos, 2015). Using the results of these two analyses, we divided our data set into two categories, neutral SNPs and SNPs under divergent selection. We used the neutral SNPs to calculate values of pairwise *F*_ST_.

To identify SNPs associated with environmental parameters, we used BayeScEnv (Villemereuil & Gaggiotti, 2015), an approach similar to BAYESCAN, but which aims to detect outlier loci associated with environmental parameters. BayeScEnv computes posterior probabilities of three models: a neutral model, a locus‐specific model, and a local adaptation model linked to the environmental variable (Villemereuil & Gaggiotti, 2015). For this approach, we used all the SNPs 15,307 (neutral and under selection), and we used only mean annual precipitation (BIO12) as predictor variable. We ran BayeScEnv 10 times and averaged results over the 10 independent runs. We used the default parameters recommended for long runs to achieve convergence of MCMC (Foll & Gaggiotti, 2008; Villemereuil & Gaggiotti, 2015) and used a false discovery rate of 0.05 to reduce the number of false positives (Foll & Gaggiotti, 2008).

As an alternative approach to confirm if the outlier loci identified by BayeScEnv were consistent among methods, we used the latent factor mixed models (LFMM; Frichot, Schoville, Bouchard, & François, 2013), which measures the associations between genotype and phenotype or environmental variables while accounting for underlying population structure. We ran five separate MCMC runs with a latent factor of *K* = 1, based on preliminary structure and PCA, and using mean annual precipitation (BIO12). We used only average bill length for the phenotype–genotype association, as bill length and bill height were highly related (*R* = 0.8557, *p* = <2.2 e^−16^). *p*‐Values from all five runs for the two independent genomewide association tests were combined and adjusted for multiple tests using a false discovery rate correction of 0.05. We used the default parameters recommended for long runs to achieve convergence of the MCMC (Frichot et al., 2013), as we did with BAYESCAN and BayeScEnv.

Finally, we also performed a redundancy analysis (RDA) using the entire set of loci to identify outlier loci and their correlation to environmental variables (Forester, Lasky, Wagner, & Urban, 2018). As explanatory variables in the redundancy analysis, we used mean annual temperature (Bio1), temperature seasonality (Bio4), mean annual precipitation (Bio12), and precipitation seasonality (Bio15). Once obtained the first three axes from the RDA, we performed an outlier identification process by assuming that outlier loci were located above or below three *SD*s from the mean of the empirical distribution given by the scores of each axis. Finally, we correlated the observed allele frequency across populations with each of the four environmental variables to determine which was the environmental variable that explained the largest amount of variance in the structure of those outlier loci (Forester et al., 2018).

### 2.5 Candidate gene identification

We used the outlier loci identified by all four methods (*p* < .08) and aligned the sequences to the reference genome of Zebra finch (*Taeniopygia_guttata*‐3.2.4 reference Annotation Release 103 NCBI (https://blast.ncbi.nlm.nih.gov/Blast.cgi), using the basic alignment search tool (Blast) from NCBI to align the sequences. We used “**nr/nt**” database and with setting parameters max target sequences to 100, expect thresholds to 10, word size to 28 and max matches in a query to 0. We considered a locus homologous if the e‐value returned was smaller than 1.0 e^−10^. We determined whether there was any gene within 25 kb upstream or downstream of each candidate SNP to focus on genes likely to be within the same linkage group as our SNP (Bay et al., 2018). Then, we calculated the allele frequencies for each SNP under selection that was linked to a candidate gene previously associated with morphological, phenotypic, and metabolic functions related to environmental variables (Bay et al., 2018). To calculate the allele frequencies, we only used the populations in which we had more than five individuals. If there was more than outlier SNP linked to the same candidate gene (e.g., BMP5), we calculated average allele frequencies across all SNPs.

### 2.6 Population structure of neutral loci

We used ADMIXTURE to evaluate population structure with different numbers of hypothetical populations (*k*). We ran ADMIXTURE ver. 1.22 (Alexander, Novembre, & Lange, 2009) using 20,000 bootstrap replicates. We used *k* values between 1 and 9 with five iterations for each value; stabilization of parameters was checked for 100k length of burn‐in and 100k MCMC simulations. To evaluate optimal partitioning in ADMIXTURE, cross‐validation (CV) error values were computed for each *k* using a fivefold cross‐validation procedure.

To determine how neutral genetic variation in our data set was distributed among populations, we conducted a principal component analysis (PCA) in the package ADEGENET 2.0.1 (Jombart & Ahmed, 2011), excluding the loci identified to be under selection by BAYESCAN and OUTFLANK (see above). We identified SNPs that were under linkage disequilibrium (LD) by using the SNPRelate software assuming a threshold of 0.5 (Zheng et al., 2012, 2017). We found that 54% of our SNPs were in LD (8,301 SNPs). Then, we performed two PCAs, one with the entire neutral loci set and the second one only with the neutral loci not under LD (7,006). Finally, we used a discriminant analysis of principal components (DAPC) as another method to identify subpopulations of the species using the ADENEGET package. We performed DAPC only with the six populations in which sample size was larger than five individuals.

Additionally, we estimated pairwise *F*_ST_ values for each locus individually and across all loci (Weir & Cockerham, 1984) excluding outlier loci as identified by BAYESCAN and OUTFLANK. We used the R package HEIRFSTAT 0.04–22 (Goudet, 2005) for all global calculations and the R package StAMPP for all pairwise calculations (Pembleton, Cogan, & Forster, 2013). As some populations had only a few individuals, we calculated pairwise *F*_ST_ values along with confidence intervals and *p*‐values between populations using 10,000 nonparametric bootstrap replicates. To understand whether geographic distance or environmental distance among populations influences population structure (i.e., isolation by distance [IBD] and isolation by environment IBE), we correlated coastal and precipitation distances to pairwise *F*_ST_ values using a multiple matrix regression implemented in the ECODIST package (Goslee & Urban, 2007; Lichstein, 2007; Wang, 2013). Significance was assessed using 10,000 permutations of the distance matrices. *F*_ST_, coastal and precipitation distance matrices were scaled prior to analysis by subtracting the mean and dividing it by the standard deviation of the data set. Scaling of the predictor variables allows for direct comparison of the regression coefficients in order to understand the relative contribution of each independent variable over genetic distance. Since Mangrove Warbler is a species restricted to Mangrove habitats, we believe that coastal distance between populations is a good proxy for dispersal distance and thus did not consider Euclidean distance in the Matrix Regression. Analyses in R were performed using version 3.5.2 (R Core Team, 2018).

### 2.7 Phenotypic variability and traits under selection

To determine patterns of morphological variation along the gradient, we performed regressions for each trait against precipitation. It is possible that changes in morphological traits are the result of allometry between body mass and other traits. To account for this, we also regressed the residuals of the relationship between body mass and each trait against precipitation. We also reduced the five morphological traits into two principal components (PC). Finally, we fitted a smooth surface using precipitation (BIO12) and salinity (ppm) as dependent variables and the morphological PC as coordinates for fitting. The regressions and principal component analysis were performed in R, and for fitting the smooth surface, we used ordisurf function in the VEGAN v.2.0‐10 package (Oksanen et al., 2016).

To assess the level of phenotypic structure in our data, we compared neutral genetic differentiation (*F*_ST_) to phenotypic differentiation (*P*_ST_). To calculate *P*_ST_, we first estimated within‐population and among‐population variance using a linear mixed model with only intercept as a fixed effect and populations as random effects. We then used the within‐population variance as the residual variance and between‐population variance as the variance of the random effect. Confidence intervals of the within‐ and between‐population variances were estimated using 1,000 parametric bootstrap replicates (Leinonen, McCairns, O'Hara, & Merilä, 2013). The variances and confidence intervals were estimated using the lmer and confint functions in the lme4 package (Bates, Maechler, Bolker, & Walker, 2015).

Using the estimated within‐ and between‐population variances, we quantified the phenotypic divergence in a trait across populations using *P*_ST_ for each morphological trait (except body mass; Brommer, 2011).

$$P_{\text{ST}}=\frac{\frac{c}{h^{2}}\sigma_{B}^{2}}{\frac{c}{h^{2}}\sigma_{B}^{2}+2\sigma_{W}^{2}}$$

In this equation, $\sigma_{B}^{2}$ and $\sigma_{W}^{2}$ are the between‐ and within‐population phenotypic variances, respectively, *h* ^2^ is the heritability of the trait under study, and the scalar *c* expresses the proportion of the between‐population variance that is due to genetic effects across populations (Brommer, 2011). Under controlled conditions, phenotypic differences should be entirely due to additive genetic effects, so *c*/*h* ^2^ = 1 and *P*_ST_ is equivalent to *Q*_ST_, and analogous to *Q*_ST_ for a given quantitative trait (Wright, 1950). In wild populations, *h* ^2^ and *c* are usually difficult to estimate (Brommer, 2011) and nonadditive genetic effects such as selection can strongly influence the estimation of *P*_ST_ (Brommer, 2011; Brommer, Hanski, Kekkonen, & Väisänen, 2014; Leinonen, Cano, Mäkinen, & Merilä, 2006; Pujol, Wilson, Ross, & Pannell, 2008). Consequently, we used a sequence of 100 values of *c*/*h* ^2^ between zero and two (Brommer, 2011). The objective of this approach is to estimate the value of *c*/*h* ^2^ for which *P*_ST_ is larger than *F*_ST_. The smaller the critical value of *c*/*h* ^2^ for which *P*_ST_ is larger than *F*_ST_, the more likely it is that selection influences morphological trait evolution. The critical *c*/*h* ^2^ value thus reflects the robustness of the comparison between *P*_ST_ and *F*_ST_ (Brommer, 2011). As *c*/*h* ^2^ approaches 1, the morphological trait is assumed to evolve under neutral conditions. We repeated this procedure for each phenotypic trait measured in the field since all of them have been shown to be heritable in multiple bird species (Charmantier, Kruuk, Blondel, & Lambrechts, 2004; Teplitsky, Robinson, & Merilä, 2014). For interpretation, we use the value of *c*/*h* ^2^ for which *P*_ST_ is larger than *F*_ST_ computing *P*_ST_ using the lower boundary of the confidence interval of $\sigma_{B}^{2}$ and $\sigma_{W}^{2}$.

## 3 RESULTS

### 3.1 Genomic signals of selection

**Figure 2:**
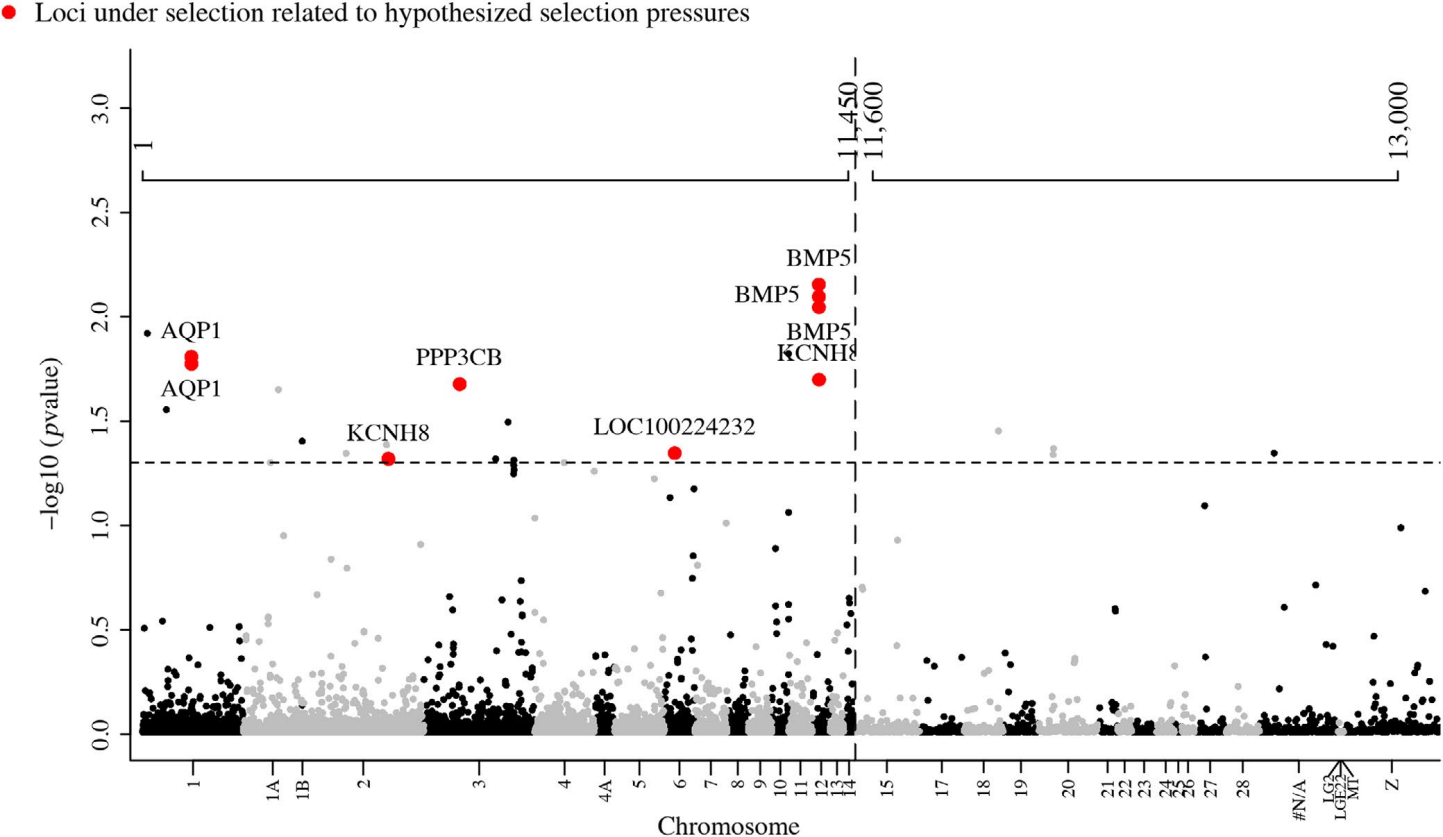
Manhattan plots showing the significance level (FDR‐corrected) for SNP associations with precipitation of the mean annual precipitation (BIO12). The horizontal dashed line represents *p* = .05 and the vertical dashed line represents a change in plot scale. Colors distinguish different chromosomes and red points are SNPs that codified for candidate genes relate with osmoregulation and bill morphology. Axis at the top of the figure refers to the position of the loci in the alignments

After filtering, we obtained 15,307 SNPs (Table S1, Appendix S1). We removed outlier loci identified with BAYESCAN (20) and OUTFLANK (25) such that 15,262 putatively neutral loci remained to compute pairwise *F*_ST_. Using BayeScEnv and LFMM, we identified a total of 38 outlier SNPs associated with precipitation (BIO12) from the original loci set: 14 SNPs were found exclusively with the BayeScEnv approach, 11 SNPs were found exclusively with LFMM, and 12 SNPs were found with both methods (Figure 2, Table 2). In some cases, multiple SNPs mapped to the same locus. From the 38 SNPs, we identified 19 genes; these include functions such as supplying calcium to cardiac muscle (RYR2), neural regulation (NRG3), different cell processes (CCSER1, DDX10), GTPase activation (RIN3), protein kinase activation (LOC100229672), and transmembrane proteins (CCDC91). The strongest associations (lowest *p* value) between genotype and precipitation were upstream of genes with known function in avian morphology and osmoregulation (Figure 2). For osmoregulation, the strongest associated genes were a sodium/chloride exchanger (LOC100224232), potassium channel regulators (KCHN7, KCHN8), and aquaporin 1 (AQP1) (Figure 2, Table 2). In addition, we found strong associations with a candidate gene with known function in avian morphology, bone morphogenetic protein (BMP5), which plays a key role in bone and cartilage development (Figure 2, Table 2).

**Table 2:** Genes identified to be associated with precipitation, their scaffold, and position (in base pairs) according to the Yellow Warbler genome and the chromosome location according to the Zebra Finch genome

Scaffold	Position	Chr	LFMM (bill size)	LFMM (BIO12)	BayeScEnv	Genes in region
Scaffold1113	210951	3	0.035	0.029		RYR2
Scaffold1195	192695	20	0.047	0.03		Unknown
Scaffold117	1149075	unknown		0.041	0.042	Unplaced genomic scaffold
Scaffold12801	8966	27		0.082		LOC105759198
Scaffold1318	167838	12	0.0012	0.001	0.008	BMP5^a^
Scaffold1318	167838	12	0.007	0.008	0.009	BMP5^a^
Scaffold1318	167838	12	0.006	0.007	0.006	BMP5^a^
Scaffold139	1067066	1		0.014	0.016	GHRHR, AQP1^a^
Scaffold139	1067066	1		0.018	0.017	GHRHR, AQP1^a^
Scaffold1256	299234	2			0.045	Unknown
Scaffold1256	299234	20			0.045	Unknown
Scaffold139	1067066	18		0.035		Unknown
Scaffold230	836124	3		0.021	0.019	TRPS1, KCNH8^a^
Scaffold139	1067066	2	0.048	0.045		Unknown
Scaffold139	1067066	2	0.046			Unknown
Scaffold1472	146957	1			0.028	DDX10
Scaffold171	1083633	2		0.047	0.045	KCNH7^a^
Scaffold1	5275185	6		0.042	0.040	PPP3CB^a^
Scaffold288	1430426	5		0.053	0.05	RIN3
Scaffold386	734965	6	0.065	0.058		Unknown
Scaffold386	734965	6	0.068			Unknown
Scaffold29	2030764	3			0.05	LOC100228354
Scaffold29	2030764	3			0.051	LOC100228354
Scaffold29	2030764	3			0.048	LOC100228354
Scaffold29	2030764	3			0.054	LOC100228354
Scaffold40	1908337	1A	0.052	0.048		NRG3
Scaffold338	1100876	4		0.059		CEP85L
Scaffolf37	328018	7		0.085		MAPK10
Scaffold444	758601	3	0.048	0.051		CCSER1
Scaffold450	908560	10	0.069	0.065		CCDC91
Scaffold426	565841	1B			0.038	LOC100229672
Scaffold4515	29740	10			0.08	Unknown
Scaffold4806	52910	12		0.021	0.020	LOC100224232^a^
Scaffold575	437602	4			0.05	Unknown
Scaffold663	532755	10			0.015	Unknown
Scaffold663	532755	1A			0.022	Unknown
Scaffold663	532755	1			0.012	Unknown
Scaffold663	532755	Z			0.080	Unknown

Note

LFMM (Bill Size), LFMM (BIO12), and BayeScEnv columns show FDR‐corrected *p*‐values (*p* < .08) for each analysis identifying these loci as outliers. Coding genes within 25 kb up or downstream of the SNP are listed in Genes in region column.

a Loci that support our hypothesis.

**Figure 3:**
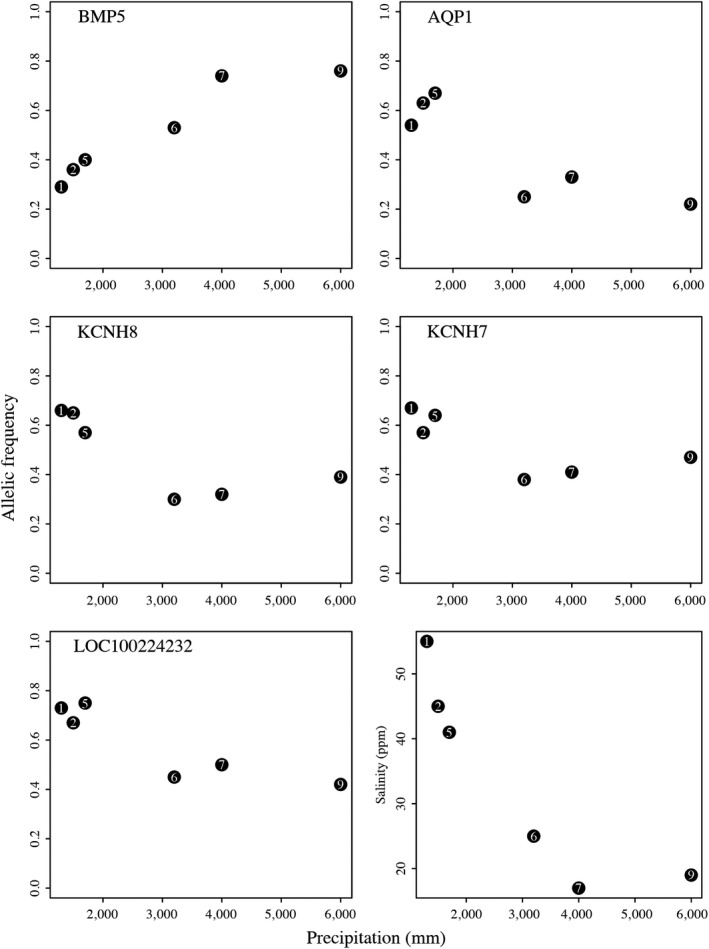
Allelic frequencies of outlier loci related to osmoregulation and bill size by population in relation to precipitation. We also show the relationship between precipitation and salinity for reference. Functions related to the loci in the table are as follows: AQP1, Aquaporine; BMP5, Bone Morphogenic Protein; KCHH8, Subfamily H. Opening and closing of K^+^ and Na^+^ Channels, KCHN7 = Subfamily H. Opening and closing of K^+^ and Na^+^ Channels, LOC100224232 = Na^+^ transporting protein. Site column represents the geographic position of localities as shown in Figure 1. Only six populations are shown since we excluded populations with less than five individuals for this analysis. Populations are as follows: 1 = Naranjo, 2 = Tamarindo, 5 = Chomes, 6 = Tarcoles, 7 = Sierpe, 9 = Golfito

We identified a total of 12 SNPs that were associated with bill size using the LFMM: two SNPs were found exclusively with this approach, while the other 10 SNPs were also identified in previous analyses with precipitation using either BayeScEnv or LFMM (Figure 2, Table 2). From the 12 SNPs identified, seven genes had known functions including supplying calcium to cardiac muscle (RYR2), neural regulation (NRG3), cell processes (CCSER1), transmembrane proteins (CCDC91). Bone morphogenetic protein (BMP5) showed the strongest associations between genotype and phenotype, similar to what we found in our genotype–environment association (Figure 2, Table 2). We found that variation in allele frequencies followed environmental changes in precipitation (Figure 3).

Complementary, the first three axes of the redundancy analysis explained 84% of the variability in SNP loci. Although the correlation with environmental variables was not significant (*p* > .05), the first axis was positively related to Bio4 and the second and third axis were positively related with Bio1, Bio 12, and Bio 15 (Figure S4). The outlier analysis using the RDA scores from the first three RDA identified 136 outlier SNPs that were putatively associated with BIO1, BIO4, BIO12, BIO15. From the 136 SNPs, 30 loci were located in genes with functions related to avian bone morphology and osmoregulation. For osmoregulation, we found genes associated with kidney development (NPNT), sodium exchanger (LOC100232644, SLC4A11, LOC100217927, LOC100221646), potassium channel regulators (KCNQ3, KCTD2, LOC100231406, KCTD5), and aquaporin 4 (AQP4) (Table S2; Figure S4). In addition, we found candidate genes with known function in avian bone morphology (BMP1, COL21A1, LOC105759070, LOC100226932), which could play roles in the development of bone and cartilage (Table S2, Figure S4).

### 3.2 Population structure

Pairwise *F*_ST_ using putatively neutral loci ranged from 0.005 between two populations in the same habitat (drier end of the gradient) to 0.057 between two populations in different habitats (at the drier and wetter ends of the gradient), but none of the pairwise *F*_ST_ values were significant (*p* > .05). The mean pairwise *F*_ST_ was 0.015 ± 0.09 (Table 3). The *F*_ST_ values were too low (<0.10) for reliable estimation of migration rates, which suggests ongoing gene flow among *S. p. xanthotera* populations (Meirmans, 2014). Additionally, it did not observe significant population structure from ADMIXTURE analysis (Figure S1, Appendix S1). The best fitting resolution according to the calculation of CV errors was *k* = 1 (Figure S2, Appendix S1). Multiple matrix regression showed no influence of coastal or precipitation distance and genetic distances among populations suggesting little influence of isolation by distance and isolation by environment at least on neutral loci (*R* ^2^ = 0.06, *F* = 1.1, *p* = .36; coastal distance, *β* = 0.003, *p* = .5; precipitation distance, *β* = −0.006; *p* = .17).

**Table 3:** Pairwise *F*_ST_ between each population pair sampled in the study

	1	2	3	4	5	6	7	8
1	—	0.011	0.04	0.08	0.03	0.018	0.015	0.005
2		—	0.05	0.02	0.006	0.057	0.02	0.05
3			—	0.049	0.042	0.023	0.033	0.006
4				—	0.02	0.05	0.007	0.016
5					—	0.02	0.008	0.02
6						—	0.015	0.036
7							—	0.003
8								—
9								0.014

Note

Population names correspond to 1 = Naranjo, 2 = Tamarindo, 3 = Junquillal, 4 = Chira, 5 = Chomes, 6 = Tarcoles, 7 = Sierpe, 8 = Osa, 9 = Golfito. *F*_ST_ was assessed using 1,000 nonparametric bootstrap replicates. None of the *F*_ST_ values were significant; *p* > .05.

We found high congruence between the lack of support of population structure from ADMIXTURE and the clustering pattern by PCA, which suggested no distinct population clustering (Figures S1 and S2). The populations of Mangrove Warblers did not cluster geographically or by environment according to their scores on the first two axes (Figure S3, Appendix S1), which accounted for 4.2% and 3.8%, respectively, of the observed genetic variation. In addition, the PCA using only loci that are not in LD did not find distinct population clustering, with PC1 and PC2 accounting for 7.6% and 8.2% of genetic variation (Figure S3). Also, the DAPC analysis did not identify any clusters within the data (Figure S4, Appendix S1).

### 3.3 Phenotypic trait differentiation and the role of environmental factors

**Figure 4:**
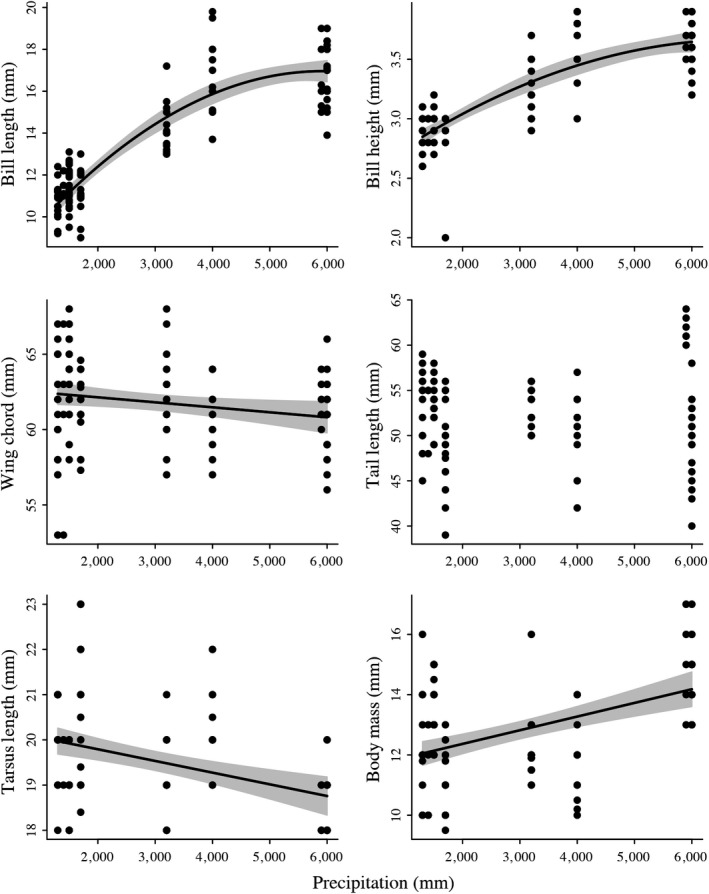
Relationship between morphological traits and mean annual precipitation (BIO12) in individuals of Mangrove Warbler. Solid black line represents the estimated model and grey polygon shows de 95% confidence interval of the regression. Panels with no regression lines indicate that the regression was not significant

**Figure 5:**
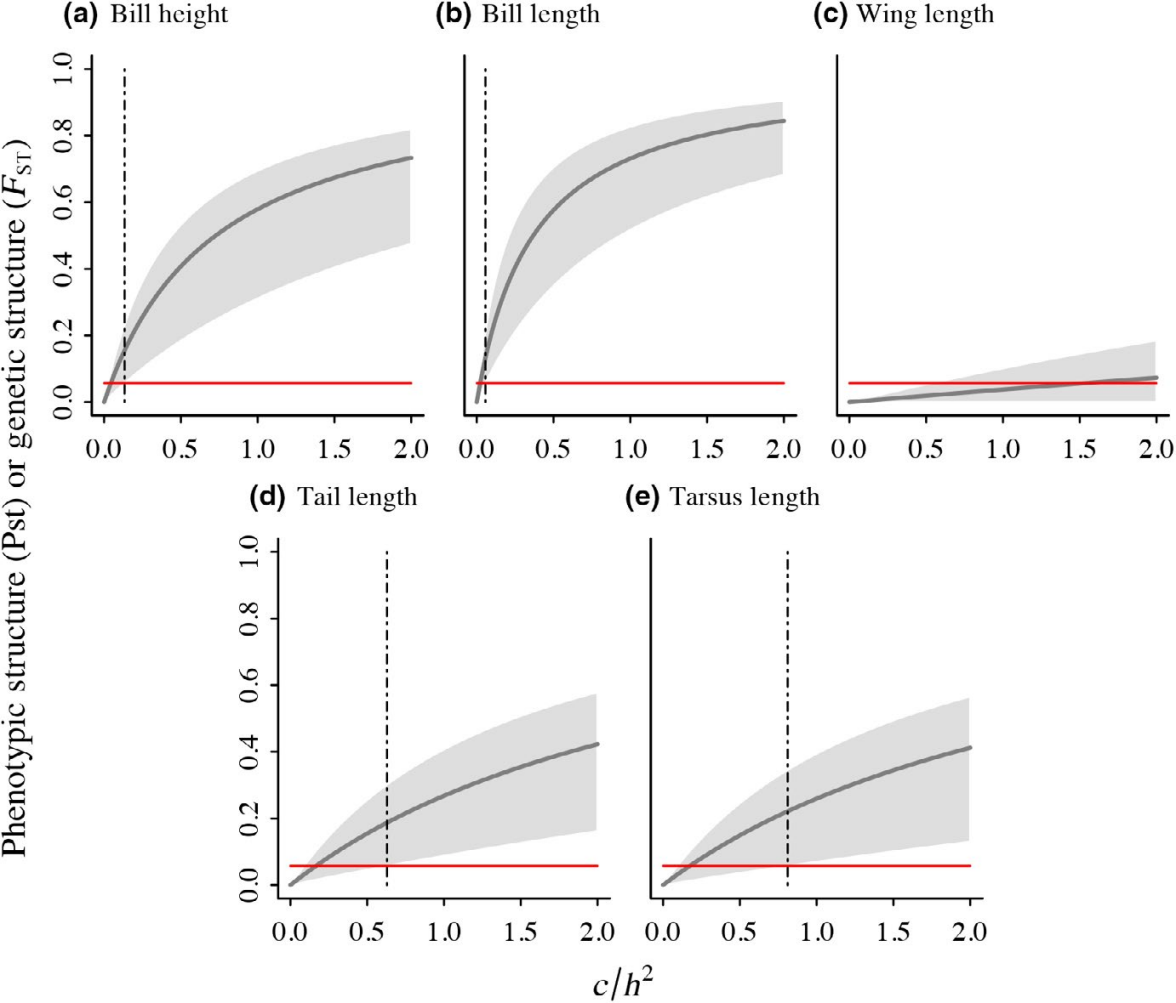
*P*_ST_–*F*_ST_ comparisons of phenotypic differentiation with neutral genetic differentiation. Our results were consistent with divergent selection on bill height (a) and bill length (b) but not on wing length, tail length and tarsus length (c–e). *P*_ST_ (black line) is plotted as a function of *c*/*h* ^2^ (*x*‐axis) with CIs (dotted black line and grey interior). The value of *c*/*h* ^2^ at which the lower confidence limit of *P*_ST_ (lower dotted black line) equals the global *F*_ST_ (horizontal red line) is the critical value of *c*/*h* ^2^ (vertical black dashed line) at which *P*_ST_ no longer exceeds *F*_ST_. Lower critical value represent more robust inferences of selection are to environmental effects. *Y*‐axis shared by unit‐less *P*_ST_ and *F*_ST_, which vary between zero and one. *X*‐axis is *c*/*h* ^2^, which represents the degree to which the overall phenotypic divergence among populations relative to within populations is due to additive genetic effects

We found that bill height, bill length, body mass, wing length, and tibia length were significantly related to mean annual precipitation (Figures 1b and 4; Table 4). Bill height and length increased with mean annual precipitation while wing length, tibia length, and body mass decreased as precipitation increased (Figure 4). We found similar results when accounting for the allometric relationship between body mass and other phenotypic traits, so we present only the raw data. The principal component analysis showed that the first PC was related mostly to bill morphology (loadings: bill height = 0.6, bill length = 0.59), explaining 41.2% of the variation, and the second component was mostly related to wing and tail length (loadings: wing length = −0.58, tail length = −0.71) and explained 26.4% of the variation. Tibia length was mostly related to the third PC (loading = −0.94) and thus was not included in the smoothing analysis. Both smoothing surfaces included a significant coefficient (Figure 1b; precipitation: *F* = 90.5, *p* < .001; salinity: *F* = 48.9, *p* < .001) and explained a considerable amount of variability in the dependent variable (precipitation: *r* ^2^ = .88; salinity: *r* ^2^ = .79). The fitting of the smooth surface on the phenotypic data showed that changes in the environment were mostly related to changes in bill morphology which was consistent with the individual analyses performed (Figures 1b and 4). *P*_ST_/*F*_ST_ comparisons revealed that phenotypic differentiation (*P*_ST_) was higher than *F*_ST_ in two (bill height and bill length) of the six traits evaluated (Figure 5, Table 5). The critical value of *c*/*h* ^2^ for bill length was 0.05, and for bill height, it was 0.13 (Figure 5, Table 5).

**Table 4:** Significant regression parameters of the relationship between morphological traits and precipitation (BIO12) among populations of Mangrove Warbler

Trait	Intercept	*x*	*x* ^2^	*p*	*R* ^2^
Bill length	6.6	0.004	−3 × 10^−7^	<.001	0.8
Bill height	2.4	0.09	−3 × 10^−8^	<.001	0.7
Wing chord	62.8	−3 × 10^−4^		.03	0.03
Tarsus length	20.3	−2.6 × 10^−4^		<.01	0.13
Body mass	11.5	4.5 × 10^−4^		<.001	0.2

Note

Intercept, *x* and *x^2^* columns represent the regression coefficients and *p* shows the significance of the regression.

**Table 5:** Results of *P*_ST_–*F*_ST_ comparisons for bill length, bill height, wing length, tail length, and tarsus

Trait	$\sigma_{B}^{2}$	$\sigma_{W}^{2}$	*P*_ST_	*c*/*h* ^2^
Bill length	0.01 (0.002, 0.03)	0.003 (0.002, 0.005)	0.91 (0.60, 0.98)	0.13
Bill height	0.04 (0.009, 0.08)	0.006 (0.005, 0.008)	0.87 (0.42, 0.97)	0.05
Wing length	0.0001 (0, 0.0005)	0.002 (0.001, 0.003)	0.32 (0.07, 0.78)	Inf
Tail length	0.004 (0.0004, 0.009)	0.005 (0.004, 0.007)	0.18 (0.11, 0.89)	0.84
Tarsus	0.002 (0.0002, 0.004)	0.002 (0.001, 0.003)	0.68 (0.14, 0.91)	0.82

Note

The modes of the between ($\sigma_{B}^{2}$)‐ and within ($\sigma_{W}^{2}$)‐population variance components are followed in parentheses by the 95% confidence intervals from the posterior distribution of a Bayesian generalized linear mixed model. *P*_ST_ values for each trait are computed at the null assumption of *c*/*h* ^2^ = 1 with 95% confidence intervals in parentheses. *c*/*h* ^2^* is the critical value of *c*/*h* ^2^ at which the lower confidence interval for *P*_ST_ exceeds the global *F*_ST_. Lower critical values represent more robust inferences of environmental selection.

## 4 DISCUSSION

Using an integrative approach, we found that Mangrove Warbler populations along the precipitation/salinity gradient of the Pacific coast of Costa Rica maintain significant phenotypic divergence despite the absence of genetic structure across most of the genome, suggesting high gene flow among populations. According to the four objectives of our study, our results show that (a) genes associated with bill growth and osmoregulatory pathways are associated with precipitation, (b) there is extremely low genetic structure in neutrally evolving loci, (c) morphological traits (bill size) change significantly along the gradient, and (d) bill phenotypic differentiation (*P*_ST_) is substantially higher than genetic differentiation (*F*_ST_). All these evidence points to the hypothesis that divergent natural selection at the gradient's extremes may be strong enough to counteract the homogenizing effect of gene flow potentially promoting initial steps of ecological speciation.

### 4.1 Candidate genes at outlier loci

Combining all analysis used to identify outlier loci, we found 23 candidate genes related to osmoregulation processes. Some of these candidate genes are specialized to activate sodium–potassium channels which could help shed the excess inorganic ions and retain water (Maley, 2012). The candidate genes AQP1 and AQP4 code for aquaporin protein types 1 and 4. Aquaporins (AQPs) are a family of transport proteins that confer high‐membrane water permeability in various tissues in animal, plants, and microorganisms. In chickens, for example, AQPs play a major role in regulating the total body water balance by concentrating or diluting uric acid (Sugiura et al., 2008). In addition, the candidate gene NPNT is related to kidney development, which is the key organ associated with osmoregulation in passerine birds (Sabat et al., 2004). These outlier genes known to be involved in osmoregulation suggest that salt regulation and water availability may pose a challenge to Mangrove Warblers and potentially other passerine insectivorous species in the Pacific coast of Costa Rica. This physiological divergence in contrasting environments has also been found in a number of taxa, including killifish (Fuller, Mcghee, & Schrader, 2007; Whitehead & Crawford, 2006), sunflowers (Karrenberg, Edelist, Lexer, & Rieseberg, 2006), and other bird species (Maley, 2012).

The candidate genes BMP1 and BMP5 are thought to be involved in evolution of avian bill shape (Abzhanov, Protas, Grant, Grant, & Tabin, 2004; Badyaev, Young, Oh, & Addison, 2008). In addition, the candidate genes COL21A1, LOC105759070, LOC100226932 are involved in cartilage development, which could be related to bill morphology. Comparisons of *P*_ST_/*F*_ST_ indicated that variation found in bill size cannot be explained by genetic drift alone. These results along with the association between BMP and environment suggest that observed bill morphology variation in Mangrove Warbler individuals along the gradient may be the result of natural selection (Figure 3).

Although it is well known that bill size is highly heritable in birds (Eroukhmanoff et al., 2013; Grant & Weiner, 1999; Maley, 2012; Ricklefs, 2012), *P*_ST_ was calculated only from phenotypic data and thus we cannot disentangle the contribution of plasticity and genetic variation in the observed trait variability. Since most documented phenotypic traits are affected by environmental conditions, at least some fraction of variation in bill size could be due to phenotypic plasticity. To measure the exact contribution of the additive genetic differentiation to bill size, it would be essential to calculate *Q*_ST_ under common garden conditions (Pujol et al., 2008). Such experiments, however, are unfeasible to perform in most birds and especially on an endemic endangered subspecies such as Mangrove Warbler.

We acknowledge that we do not provide any direct evidence to suggest that phenotypic differences confer fitness advantages to individuals in different environments. While we have identified candidate loci associated with differences in the environment, we cannot conclude that local adaptation causes phenotypic differences reported here. Studying the reproductive output associated with phenotype and climatic conditions could be one of the useful approaches to test for fitness differences in this species. This requires, however, an exhaustive fieldwork which we were not able to perform at the time of this study.

### 4.2 Phenotypic divergence in the presence of gene flow

The strong phenotypic divergence observed along the gradient contrasts with the lack of genetic structure. Similar results have been obtained in marine organisms where high dispersal distances lead to high levels of gene flow (Fuller et al., 2007; Whitehead & Crawford, 2006). Other studies in birds have also reported strong phenotypic differentiation with gene flow mainly along elevational gradients (Cheviron & Brumfield, 2009; Milá et al., 2009; Seeholzer & Brumfield, 2018). Fewer studies, however, show phenotypic differentiation with high levels of gene flow in birds along other types of gradients. Smith et al. (2005), for example, found significant morphological divergence in the Little Greenbul (*Andropadus virens*) along an ecological gradient in African rain forests, despite relatively high rates of gene flow among populations. Badyaev et al. (2008) found that House Finches (*Carpodacus mexicanus*) that live in desert and urban areas interbreed freely but, have significant divergence in bill traits linked to differences in foraging resources among habitats.

One of the missing links in our study is the direct estimation of migration rates or gene flow among populations. Several methods in the literature allow for direct estimation of migration from genetic data under different assumptions of the timing of divergence and strength of connectivity among populations (e.g., Bayesass, Wilson & Rannala, 2003; Migrate‐n, Beerli & Palczewski, 2010). Nonetheless, these methods are not accurate when divergence among populations is very recent and population structure is low (specifically *F*_ST_ < 0.1; Faubet, Waples, & Gaggiotti, 2007; Samarasin, Shuter, Wright, & Rodd, 2017). Since Mangrove Warbler populations are hypothesized to have recently diverged from their ancestors (Chaves et al., 2012) and that we found low population structure, we believe that methods available for estimation of the number of migrants per generation in this case are not appropriate. It is known, however, that PCA of SNP data has a genealogical interpretation allowing us to make inferences about migration and connectivity among populations (McVean, 2009). Thus, from our results we can infer that migration among Mangrove Warbler populations is high because we did not observe defined clusters in our PCA based on putatively neutral loci.

It has been previously reported that bill height and length can respond to selection driven by food resource availability (Grant & Grant, 1993, 2011). Insect size distribution is positively influenced by precipitation (Janzen & Schoener, 1968) supporting our observations of smaller bill sizes in the drier habitat. The fact that bill morphology responds in the predicted direction and that we found associations between precipitation and genes in the BMP family, support the hypothesis that morphological divergence in Mangrove Warbler populations is maintained through natural selection. Alternatively, bill size has been associated with accessibility to prey, as relative longer bills length has been associated with foraging strategies that require access to prey hidden in deeper substrate (Wright & Steadman, 2012). It is possible that higher humidity in wetter forests provides habitat for a wider variety of epiphytes which in turn provide refuge for bird prey. Thus, longer bills at the wet end of the gradient can alternatively be explained by a wider range of depths and substrates at which warblers need to search for insects.

In a phylogenetic study of the Yellow Warbler complex (Chaves et al., 2012), the authors report no morphological divergence among the populations on the Galapagos islands, even along environmental gradients. These populations are as old as Mangrove Warbler populations suggesting that selection driven by differences in precipitation, salinity, and forest structure is strong. Our results are comparable to other studies that have shown that bill size responds to intense short‐term selection and, therefore, can evolve rapidly (Boag & Grant, 1981; Eroukhmanoff et al., 2013; Smith & Dhondt, 1980).

Alternatively, there is growing evidence that avian bill morphology plays an important role in heat exchange and thermoregulation, even when this trait is strongly associated with diet and foraging niche (Grant & Grant, 1993, 2002; Symonds & Tattersall, 2010; Tattersall, Andrade, & Abe, 2009). Some studies have found that bill size may be important for heat dissipation in high humidity habitats (Friedman, Harmáčková, Economo, & Remeš, 2017; Gardner et al., 2016). Having a longer bill could also help Mangrove Warbler individuals with thermoregulation, especially at the wetter end of the gradient as humidity increases with precipitation, supporting our observations of larger bill sizes in wetter habitats.

Future studies should focus on the influence of bill divergence on song characteristics in Mangrove Warblers. Natural selection on phenotypic traits can pleiotropically cause divergence in sexually selected traits (e.g., song) which may trigger ecological speciation (Caro, Caycedo‐Rosales, Bowie, Slabbekoorn, & Cadena, 2013; Laiolo & Tella, 2006; Schluter, 2001). It is well known that bill morphology limits the pace and timing at which bird songs are delivered. Both song traits are important in interspecific recognition and sexual selection (Podos & Nowicki, 2004; Seddon, 2005; Slabbekoorn & Smith, 2000). Preliminary data on Mangrove Warblers show substantial vocal variability among individuals at the extremes of the rainfall/salinity gradient, which points to the hypothesis that these populations might be undergoing initial stages of a speciation process (Figure S5).

Based on our *P*_ST_/*F*_ST_ comparisons, we cannot argue that selection influences tarsus length, wing length, and body size. However, we did find a trend in which tarsus and wing length decreased along with precipitation. Both traits have been previously reported to respond significantly to environmental changes in other bird species (Milá et al., 2010; Pennycuick, 1968; Thomas, 1996). Shorter wings could benefit individuals in the wetter end of the gradient, as shorter wings are more efficient for maneuvering in areas with denser forest (Pennycuick, 1968; Thomas, 1996). Furthermore, Mangrove Warbler individuals in drier habitats could benefit from longer legs in order to expand the diversity of perches and foraging methods when the food is scarce (Janzen & Schoener, 1968), as longer legs allow the use of a greater variety of perches (Wright & Steadman, 2012).

## 5 CONCLUSION

Our findings highlight the importance of understanding both phenotypic and genetic variation along with their links, when examining population differentiation processes in populations which are not geographic isolated. The *Setophaga petechia* species complex has abundant geographic intraspecific variation based on plumage color and pattern (Browning, 1994). At least nine subspecies are recognized in the *aestiva* group, eighteen in the *petechia* group, and sixteen in the *erithachorides* group. Such high variability might indicate that there is incipient diversification and our study suggests that such differentiation might be caused by environmental variability. Further studies in other groups within *S. petechia* can lead to a better understanding the early stages of the formation of biological diversity in a group in which numerous populations could potentially constitute incipient or full biological species.

## CONFLICT OF INTEREST

None declared.

## AUTHOR CONTRIBUTIONS

T.C.‐P. conceived the project, collected and analyzed most of the data, and wrote the manuscript; J.P.G. contributed with data analysis and manuscript writing. R.K. and M.M.M. assisted in study design and manuscript writing. J.U.‐M contributed to build RAD‐Seq libraries. R.B. contributed with analyses of genomic data and manuscript writing.

## Data Availability

The data have been deposited with links to BioProject accession number PRJNA516063 in the NCBI BioProject database (https://www.ncbi.nlm.nih.gov/bioproject/). The morphological data have been deposited in figshare (https://figshare.com/s/ba4b0d4247430c35b451).
