# Supplementary figures and images for: Strong phenotypic divergence in spite of low genetic structure in the endemic Mangrove Warbler subspecies (Setophaga petechia xanthotera) of Costa Rica
Source: Ecol Evol. 2019 Nov 19;9(24):13902–18. doi: 10.1002/ece3.5826 (PMC6953683; doi:10.1002/ece3.5826)

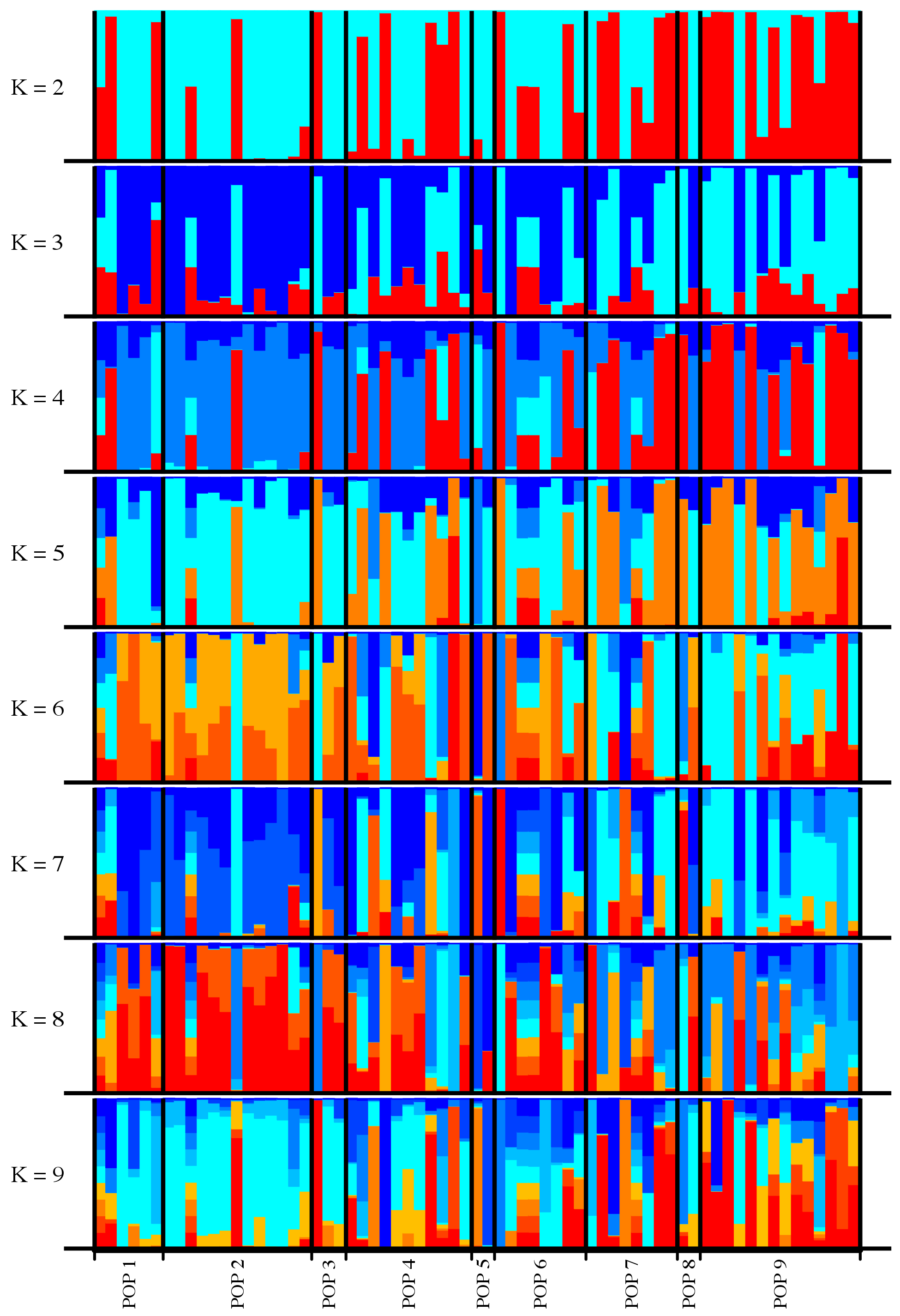

Supplement: Supplementary file 1 [file ECE3-9-13902-s001.tif]

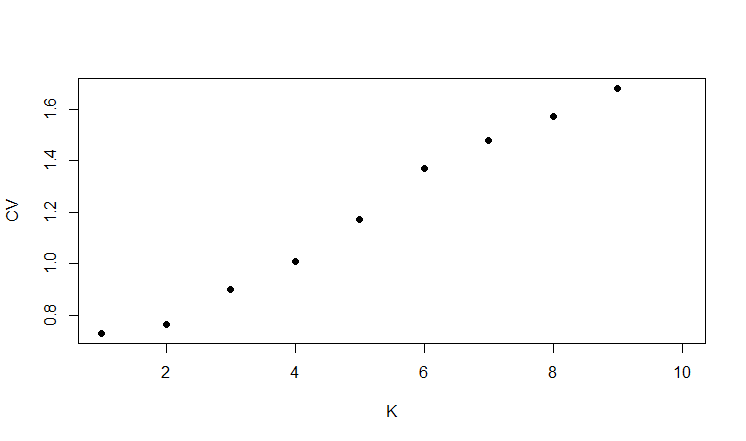

Supplement: Supplementary file 2 [file ECE3-9-13902-s002.tiff]

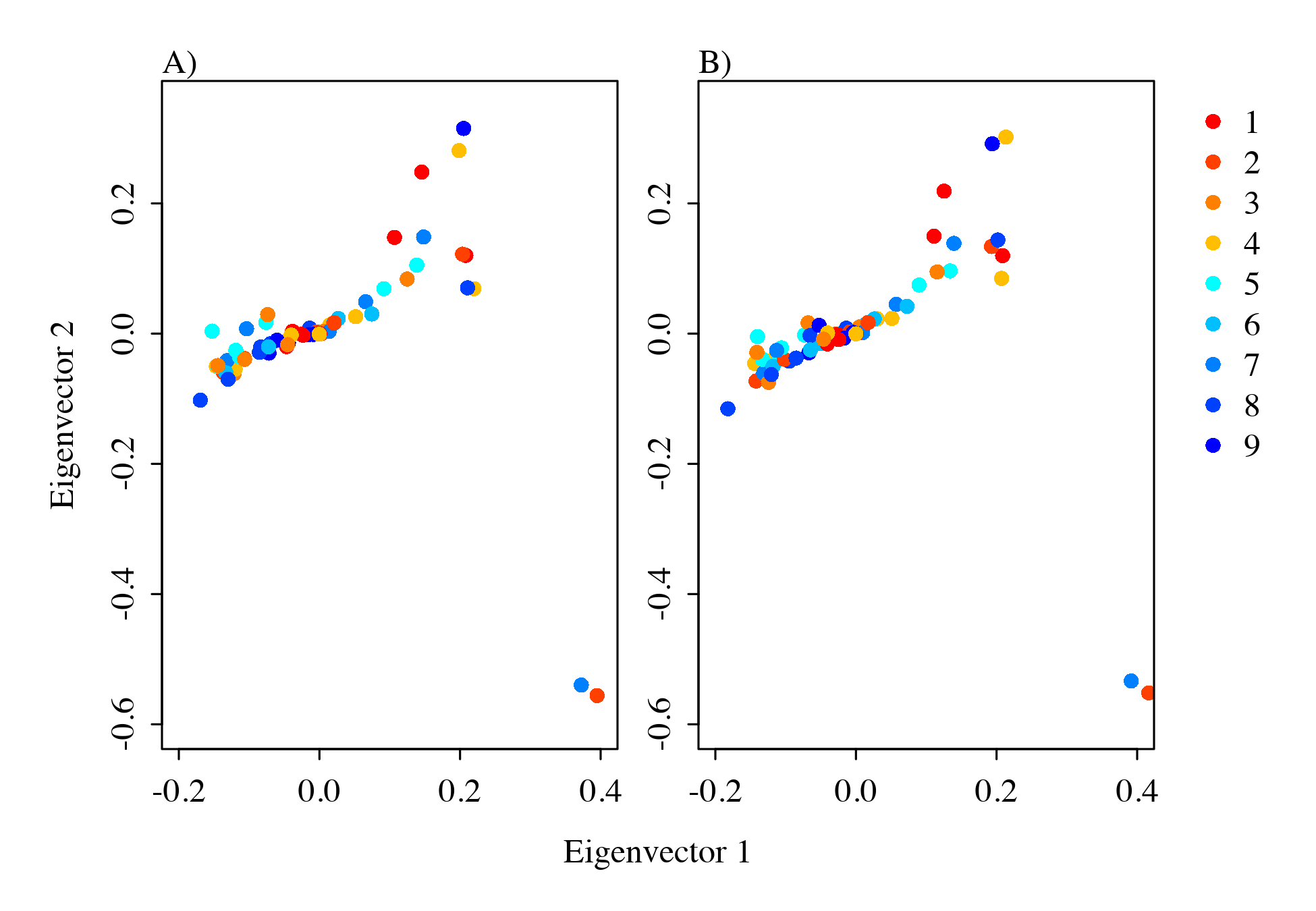

Supplement: Supplementary file 3 [file ECE3-9-13902-s003.tiff]

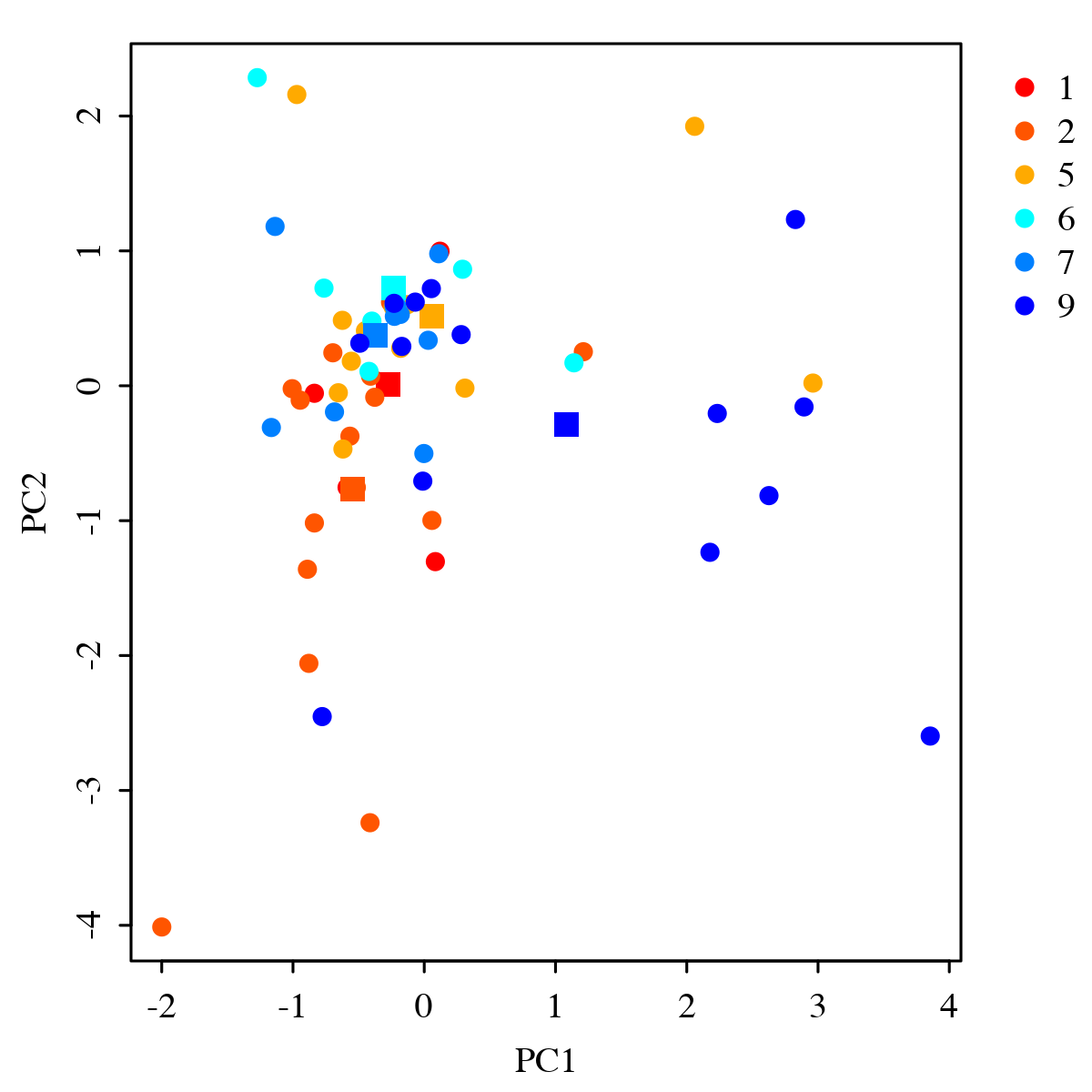

Supplement: Supplementary file 4 [file ECE3-9-13902-s004.tiff]

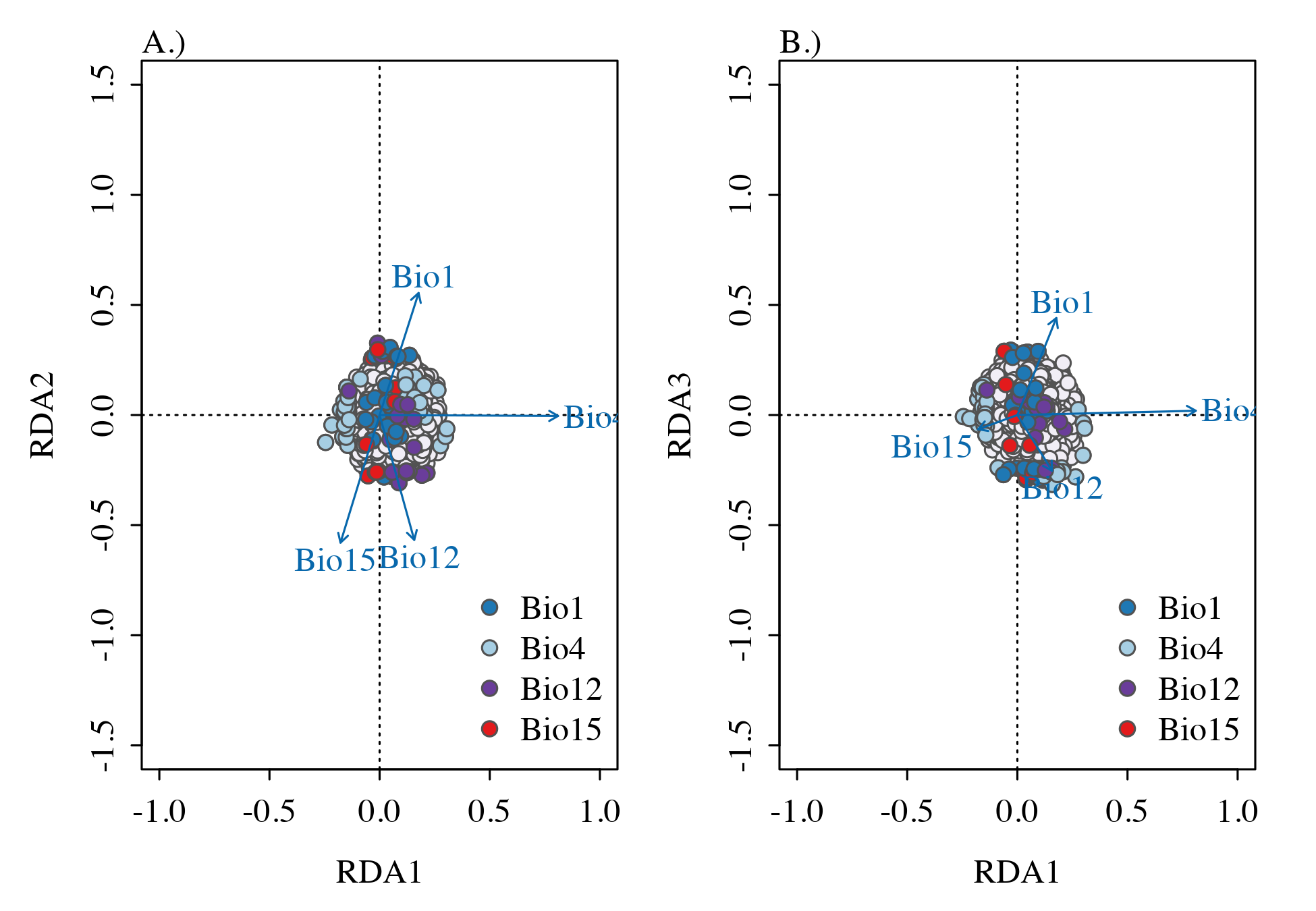

Supplement: Supplementary file 5 [file ECE3-9-13902-s005.tif]

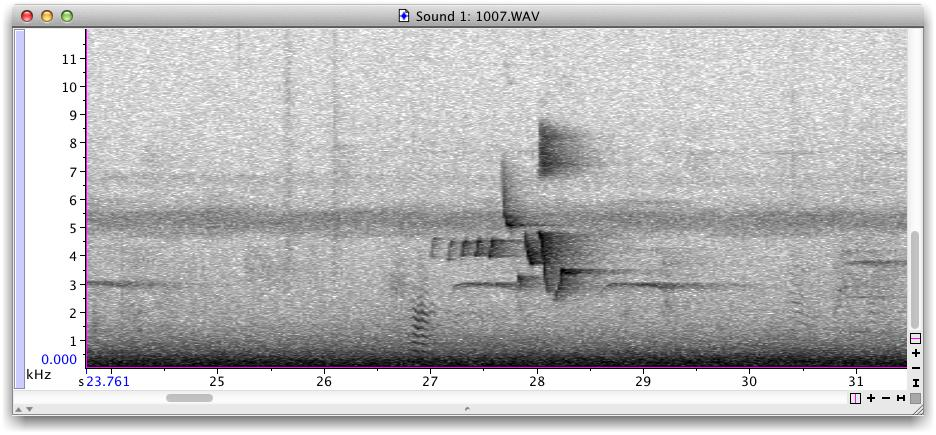

Supplement: Supplementary file 6 [file ECE3-9-13902-s006.tif]

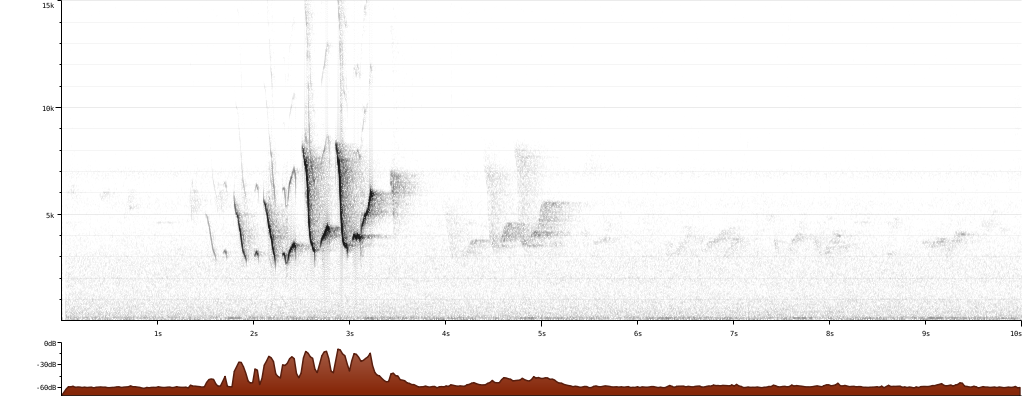

Supplement: Supplementary file 7 [file ECE3-9-13902-s007.tif]
