# Supplementary material for: Strong phenotypic divergence in spite of low genetic structure in the endemic Mangrove Warbler subspecies (Setophaga petechia xanthotera) of Costa Rica
Source: Ecol Evol. 2019 Nov 19;9(24):13902–18. doi: 10.1002/ece3.5826 (PMC6953683; doi:10.1002/ece3.5826)
Supplement: Supplementary file 8 [file ECE3-9-13902-s008.docx]

**Appendix**

Table S1. Number of SNPs retained following each filtering step. Population filters shows the refers to population’s module of STACKS and following consecutive filtering steps, we retained SNPs genotyped in at least 80% of the individuals and 80% of the sampling locations and excluded markers showing heterozygosity >0.50 within samples, MAF filters indicates removed SNPs with very low frequency, and HWE shows number of loci retained after exclusion of loci not in Hardy Weinberg equilibrium.

| Filtering Step | SNPs count |
| --- | --- |
| Stacks catalog | 1450993 |
| *Population filters* |  |
| >80% samples | 766000 |
| >80% populations | 60689 |
| *MAF filters* |  |
| >0.05 | 20490 |
| *HWE filters* |  |
| H_OBS_<0.5 | 15307 |

Table S2. Outlier loci identified by the redundancy analysis and the environmental variables with which they had the strongest correlation coefficient (R). Bio 1 = Mean annual Temperature, Bio 4 = Temperature seasonality, Bio 12 = Mean annual precipitation, Bio 15 = Precipitation seasonality. The SNP identifier, the scaffold and the position in reference to the zebra finch genome are also presented.

| SNP | Scaffold | Position | Predictor | p-value | R | Gene in regions |
| --- | --- | --- | --- | --- | --- | --- |
| 72985_105 | 1239 | 184579 | Bio1 | < 0.001 | 0.19 | FRMD4B |
| 75382_117 | 1351 | 169078 | Bio1 | < 0.001 | 0.26 | LOC100230132 |
| 7801_98 | 1414 | 154971 | Bio1 | < 0.001 | 0.35 | PCNXL4 |
| 7801_134 | 1414 | 154971 | Bio1 | < 0.001 | 0.20 | PCNXL4 |
| 77420_29 | 145 | 1569746 | Bio1 | < 0.001 | 0.20 | KIF1A |
| 77561_85 | 146 | 1902761 | Bio4 | < 0.001 | 0.19 | DNAH1 |
| 77952_75 | 148 | 1370254 | Bio1 | < 0.001 | 0.38 | SLC26A4*** |
| 56605_94 | 15 | 2313876 | Bio4 | < 0.001 | 0.42 | LOC100226932*** |
| 11827_65 | 16716 | 13133 | Bio4 | 0.001 | 0.25 | LOC105760837 |
| 12911_56 | 17427 | 5335 | Bio1 | < 0.001 | 0.24 | LOC105758916 |
| 84000_58 | 1826 | 127060 | Bio4 | < 0.001 | 0.18 | LOC105759070*** |
| 57434_57 | 182 | 938946 | Bio1 | < 0.001 | 0.26 | Unknown |
| 134253_67 | 220 | 852165 | Bio15 | < 0.001 | 0.29 | TMEM19 |
| 134253_121 | 220 | 852165 | Bio15 | < 0.001 | 0.19 | TMEM19 |
| 92781_24 | 2546 | 121344 | Bio4 | < 0.001 | 0.19 | NPNT*** |
| 93895_24 | 266 | 777548 | Bio1 | < 0.001 | 0.21 | GID8 |
| 94158_20 | 269 | 947175 | Bio4 | < 0.001 | 0.24 | TBX6L |
| 59678_23 | 372 | 906497 | Bio1 | 0.001 | 0.38 | NR2C2 |
| 24223_41 | 286 | 747144 | Bio1 | 0.001 | 0.27 | Unknown |
| 24223_108 | 286 | 747144 | Bio1 | 0.001 | 0.24 | Unknown |
| 25998_96 | 308 | 714355 | Bio1 | < 0.001 | 0.20 | Unknown |
| 26835_19 | 31 | 1767843 | Bio4 | 0.001 | 0.43 | PI4KA |
| 27268_69 | 326 | 686510 | Bio1 | 0.001 | 0.19 | BNC1 |
| 28173_43 | 33 | 1845330 | Bio1 | 0.001 | 0.19 | CEP89 |
| 100994_57 | 3476 | 43750 | Bio4 | 0.0012 | 0.18 | DFNA5 |
| 105022_113 | 4001 | 36149 | Bio4 | < 0.001 | 0.18 | CSAD |
| 32153_72 | 3 | 3342599 | Bio12 | 0.0012 | 0.21 | FBXO28 |
| 105320_86 | 4059 | 34287 | Bio4 | < 0.001 | 0.28 | KCTD9*** |
| 105320_107 | 4059 | 34287 | Bio15 | < 0.001 | 0.19 | KCTD9*** |
| 107141_72 | 434 | 798606 | Bio15 | < 0.001 | 0.19 | CBLB |
| 36381_123 | 47 | 1569407 | Bio1 | 0.001 | 0.20 | LOC100226455 |
| 63486_17 | 511 | 488975 | Bio1 | < 0.001 | 0.23 | Unknown |
| 114000_58 | 551 | 548578 | Bio12 | < 0.001 | 0.21 | Unknown |
| 115782_36 | 590 | 427514 | Bio1 | < 0.001 | 0.23 | LOC100222813 |
| 115980_22 | 596 | 423558 | Bio1 | < 0.001 | 0.22 | AQP4*** |
| 116117_88 | 59 | 1512620 | Bio12 | < 0.001 | 0.17 | Unknown |
| 116088_98 | 59 | 1512620 | Bio12 | < 0.001 | 0.24 | LOC100224008 |
| 42532_20 | 61 | 1765893 | Bio1 | 0.001 | 0.23 | Unknown |
| 64972_43 | 66 | 1425810 | Bio1 | < 0.001 | 0.23 | HOMER1 |
| 121407_8 | 727 | 343746 | Bio12 | < 0.001 | 0.23 | GPI |
| 121695_127 | 736 | 786736 | Bio4 | < 0.001 | 0.23 | Unknown |
| 65587_48 | 741 | 436833 | Bio15 | < 0.001 | 0.23 | NT5DC1 |
| 66181_117 | 820 | 505867 | Bio4 | < 0.001 | 0.25 | SGSM2 |
| 125164_98 | 830 | 333885 | Bio1 | < 0.001 | 0.32 | JPH1 |
| 127182_135 | 89 | 1382614 | Bio12 | < 0.001 | 0.26 | cyto P450 |
| 66761_65 | 8 | 4579963 | Bio1 | < 0.001 | 0.26 | BMP1*** |
| 128351_28 | 92 | 1343551 | Bio12 | < 0.001 | 0.33 | TNPO1 |
| 53533_16 | 989 | 248182 | Bio12 | 0.001 | 0.36 | Unknown |
| 69275_65 | 1075 | 552052 | Bio1 | 0.001 | 0.27 | Unknown |
| 2424_29 | 110 | 1291286 | Bio1 | 0.001 | 0.26 | KCTD2*** |
| 2424_30 | 110 | 1291286 | Bio1 | 0.001 | 0.31 | KCTD2*** |
| 5232_99 | 1268 | 179023 | Bio12 | < 0.001 | 0.30 | TTC27 |
| 5232_105 | 1268 | 179023 | Bio12 | < 0.001 | 0.30 | TTC27 |
| 7742_92 | 140 | 1065189 | Bio12 | 0.008 | 0.29 | Unknown |
| 7742_128 | 140 | 1065189 | Bio12 | 0.004 | 0.42 | Unknown |
| 9566_103 | 151 | 1535819 | Bio12 | 0.004 | 0.20 | Unknown |
| 80490_20 | 161 | 1175322 | Bio15 | 0.001 | 0.30 | LOC100221646*** |
| 80490_36 | 161 | 1175322 | Bio15 | 0.001 | 0.37 | LOC100221646*** |
| 56825_137 | 1654 | 125373 | Bio15 | < 0.001 | 0.30 | CRYAA |
| 12163_40 | 169 | 1045044 | Bio12 | < 0.001 | 0.40 | HCN1 |
| 13728_37 | 17 | 3539778 | Bio1 | 0.001 | 0.41 | Unknown |
| 83981_74 | 1825 | 114137 | Bio1 | 0.001 | 0.41 | HERC1 |
| 16727_85 | 204 | 894713 | Bio12 | < 0.001 | 0.36 | PRMT8 |
| 23906_19 | 2829 | 31749 | Bio12 | 0.001 | 0.34 | ATF1 |
| 33237_54 | 41 | 1618840 | Bio1 | 0.001 | 0.34 | LOC10022835*** |
| 33703_103 | 42 | 2724643 | Bio12 | < 0.001 | 0.24 | PTPRD |
| 107921_51 | 445 | 805235 | Bio1 | 0.001 | 0.26 | MYCBPAP |
| 107921_127 | 445 | 805235 | Bio1 | 0.001 | 0.29 | MYCBPAP |
| 109668_121 | 476 | 900277 | Bio15 | 0.001 | 0.35 | CTTNBP2 |
| 37761_109 | 501 | 499541 | Bio12 | 0.001 | 0.22 | Unknown |
| 38532_134 | 51 | 1535327 | Bio1 | 0.001 | 0.22 | LOC101233158 |
| 40694_102 | 574 | 441391 | Bio12 | < 0.001 | 0.36 | LOC100231406*** |
| 40930_16 | 57 | 1647764 | Bio12 | < 0.001 | 0.36 | GRHL2 |
| 40930_120 | 57 | 1647764 | Bio12 | < 0.001 | 0.37 | GRHL2 |
| 117829_17 | 638 | 540322 | Bio12 | < 0.001 | 0.29 | TTC13 |
| 117829_17 | 638 | 540322 | Bio12 | < 0.001 | 0.26 | TTC13 |
| 117997_100 | 640 | 735392 | Bio1 | 0.001 | 0.33 | EML6 |
| 119009_21 | 666 | 928118 | Bio1 | 0.001 | 0.30 | ZPR1*** |
| 45689_89 | 770 | 351074 | Bio12 | < 0.001 | 0.37 | KDM3B |
| 125064_59 | 82 | 2105819 | Bio1 | 0.001 | 0.30 | EDEM3 |
| 49946_44 | 850 | 288172 | Bio15 | 0.001 | 0.22 | KCTD5*** |
| 51446_93 | 901 | 385994 | Bio1 | 0.001 | 0.18 | ZNF217*** |
| 52968_26 | 967 | 416210 | Bio15 | 0.001 | 0.19 | LOC100231857 |
| 53469_10 | 9862 | 11058 | Bio12 | 0.0011 | 0.06 | LOC105759844 |
| 67908_64 | 1024 | 337617 | Bio4 | < 0.001 | 0.19 | ERN1*** |
| 130773_99 | 104 | 2065466 | Bio12 | 0.0012 | 0.14 | LOC100218844 |
| 69842_84 | 109 | 2218812 | Bio1 | < 0.001 | 0.18 | COL12A1*** |
| 54807_129 | 116 | 1213006 | Bio4 | < 0.001 | 0.14 | Unknown |
| 72037_64 | 1192 | 197402 | Bio1 | < 0.001 | 0.26 | RAB25 |
| 72985_105 | 1239 | 184579 | Bio1 | < 0.001 | 0.26 | FRMD4B |
| 75382_117 | 1351 | 169078 | Bio1 | < 0.001 | 0.28 | LOC100230132 |
| 7801_98 | 1414 | 154971 | Bio1 | < 0.001 | 0.13 | PCNXL4 |
| 7801_134 | 1414 | 154971 | Bio1 | < 0.001 | 0.15 | PCNXL4 |
| 77420_29 | 145 | 1569746 | Bio1 | < 0.001 | 0.20 | KIF1A |
| 77561_85 | 146 | 1902761 | Bio4 | < 0.001 | 0.31 | DNAH1 |
| 77952_75 | 148 | 1370254 | Bio1 | < 0.001 | 0.16 | SLC26A4*** |
| 56605_94 | 15 | 2313876 | Bio4 | < 0.001 | 0.15 | LOC100226932*** |
| 11827_65 | 16716 | 13133 | Bio4 | 0.001 | 0.19 | LOC105760837 |
| 12911_56 | 17427 | 5335 | Bio1 | < 0.001 | 0.09 | LOC105758916 |
| 84000_58 | 1826 | 127060 | Bio4 | < 0.001 | 0.09 | LOC105759070*** |
| 57434_57 | 182 | 938946 | Bio1 | < 0.001 | 0.32 | Unknown |
| 134253_67 | 220 | 852165 | Bio15 | < 0.001 | 0.18 | TMEM19 |
| 134253_121 | 220 | 852165 | Bio15 | < 0.001 | 0.30 | TMEM19 |
| 92781_24 | 2546 | 121344 | Bio4 | < 0.001 | 0.26 | NPNT*** |
| 93895_24 | 266 | 777548 | Bio1 | < 0.001 | 0.11 | GID8 |
| 94158_20 | 269 | 947175 | Bio4 | < 0.001 | 0.12 | TBX6L |
| 59678_23 | 372 | 906497 | Bio1 | 0.001 | 0.18 | NR2C2 |
| 24223_41 | 286 | 747144 | Bio1 | 0.001 | 0.18 | Unknown |
| 24223_108 | 286 | 747144 | Bio1 | 0.001 | 0.27 | Unknown |
| 25998_96 | 308 | 714355 | Bio1 | < 0.001 | 0.26 | Unknown |
| 26835_19 | 31 | 1767843 | Bio4 | 0.001 | 0.15 | PI4KA |
| 27268_69 | 326 | 686510 | Bio1 | 0.001 | 0.25 | BNC1 |
| 28173_43 | 33 | 1845330 | Bio1 | 0.001 | 0.30 | CEP89 |
| 100994_57 | 3476 | 43750 | Bio4 | 0.0012 | 0.14 | DFNA5 |
| 105022_113 | 4001 | 36149 | Bio4 | < 0.001 | 0.16 | CSAD |
| 32153_72 | 3 | 3342599 | Bio12 | 0.0012 | 0.15 | FBXO28 |
| 105320_86 | 4059 | 34287 | Bio4 | < 0.001 | 0.23 | KCTD9*** |
| 105320_107 | 4059 | 34287 | Bio15 | < 0.001 | 0.23 | KCTD9*** |
| 107141_72 | 434 | 798606 | Bio15 | < 0.001 | 0.14 | CBLB |
| 36381_123 | 47 | 1569407 | Bio1 | 0.001 | 0.21 | LOC100226455 |
| 63486_17 | 511 | 488975 | Bio1 | < 0.001 | 0.11 | Unknown |
| 114000_58 | 551 | 548578 | Bio12 | < 0.001 | 0.23 | Unknown |
| 115782_36 | 590 | 427514 | Bio1 | < 0.001 | 0.25 | LOC100222813 |
| 115980_22 | 596 | 423558 | Bio1 | < 0.001 | 0.12 | AQP4*** |
| 116117_88 | 59 | 1512620 | Bio12 | < 0.001 | 0.15 | Unknown |
| 116088_98 | 59 | 1512620 | Bio12 | < 0.001 | 0.19 | LOC100224008 |
| 42532_20 | 61 | 1765893 | Bio1 | 0.001 | 0.19 | Unknown |
| 64972_43 | 66 | 1425810 | Bio1 | < 0.001 | 0.19 | HOMER1 |
| 121407_8 | 727 | 343746 | Bio12 | < 0.001 | 0.36 | GPI |
| 121695_127 | 736 | 786736 | Bio4 | < 0.001 | 0.14 | Unknown |
| 65587_48 | 741 | 436833 | Bio15 | < 0.001 | 0.23 | NT5DC1 |
| 66181_117 | 820 | 505867 | Bio4 | < 0.001 | 0.27 | SGSM2 |
| 125164_98 | 830 | 333885 | Bio1 | < 0.001 | 0.24 | JPH1 |
| 127182_135 | 89 | 1382614 | Bio12 | < 0.001 | 0.13 | cyto P450 |
| 66761_65 | 8 | 4579963 | Bio1 | < 0.001 | 0.24 | BMP1*** |
| 128351_28 | 92 | 1343551 | Bio12 | < 0.001 | 0.20 | TNPO1 |

*** loci that support the hypothesis
